# Supplementary material for: Combination of BRCA deep targeted sequencing and shallow whole genome sequencing to detect homologous recombination deficiency in ovarian cancer
Source: Front Oncol. 2025 Sep 1;15:1593881. doi: 10.3389/fonc.2025.1593881 (PMC12434766; doi:10.3389/fonc.2025.1593881)
Supplement: Supplementary file 1 [file DataSheet1.pdf]

Combination of *BRCA* deep targeted sequencing and shallow whole genome sequencing to detect homologous recombination deficiency in ovarian cancer

Thien-Phuc Hoang Nguyen[1,2#], Nam HB Tran[1,2#], Tien Anh Nguyen[1,2], My TT Ngo[1,2], Anh Duong Doan[1,2], Du Quyen Nguyen[1,2], Hung Sang Tang[1,2], Duy Sinh Nguyen[1,2], Cam Tu Nguyen Thi[1,2], Thanh Thuy Do Thi[1], Hoai-Nghia Nguyen[1,2], Hoa Giang[1,2], Lan N Tu[1,2]

[1] Medical Genetics Institute, Ho Chi Minh city, Vietnam

[2] Gene Solutions, Ho Chi Minh city, Vietnam

[#]These authors contributed equally to this work

Corresponding authors:

Lan N Tu: Medical Genetics Institute and Gene Solutions, Ho Chi Minh city, Vietnam (lantu@genesolutions.vn)

Table S1. Patient demographics

**Characteristic**	77 (100%)
**Age at diagnosis (range), years**	54 (21 – 85)
>50	47 (61.0%)
≤50	30 (39%)
**Subtype (N, %)**	
High-grade serous	22 (28.6%)
Endometrioid	4 (5.2%)
Mucinous	1 (1.3%)
Unknown	50 (64.9%)
**Stage (N, %)**	
I	6 (7.8%)
II	6 (7.8%)
III-IV	65 (84.4%)

Table S2. ***BRCA*** **mutation status and wGI score of ovarian cancer patients**

**ID**	**BRCA1/2 clinically significant mutation**			**Instability score**		**HRD status**
	Mutation	VAF	Conclusion	wGI	Conclusion	
S01	ND		NEG	-16	NEG	**NEG**
S02	ND		NEG	3	NEG	**NEG**
S03	ND		NEG	1	POS	**POS**
S04	ND		NEG	3	POS	**POS**
S05	ND		NEG	-10	NEG	**NEG**
S06	ND		NEG	-7	NEG	**NEG**
S07	ND		NEG	3	NEG	**NEG**
S08	ND		NEG	2	POS	**POS**
S09	*BRCA2:* 3033Nfs*11	38%	POS	-18	NEG	**POS**
S10	ND		NEG	-12	NEG	**NEG**
S11	ND		NEG	-16	NEG	**NEG**
S12	ND		NEG	-19	NEG	**NEG**
S13	ND		NEG	11	POS	**POS**
S14	*BRCA1:*Q1779Nfs*14	70%	POS	11	POS	**POS**
S15	ND		NEG	12	POS	**POS**
S16	ND		NEG	14	POS	**POS**
S17	ND		NEG	-13	NEG	**NEG**
S18	ND		NEG	-17	NEG	**NEG**
S19	ND		NEG	-18	NEG	**NEG**
S20	ND		NEG	0	POS	**POS**
S21	ND		NEG	1	POS	**POS**
S22	*BRCA1:*R1751*	64.1%	POS	14	POS	**POS**
S23	ND		NEG	5	POS	**POS**
S24	*BRCA1:*P871Vfs*31	54%	POS	-5	NEG	**POS**
S25	ND		NEG	-5	NEG	**NEG**
S26	ND		NEG	10	POS	**POS**
S27	ND		NEG	5	POS	**POS**
S28	ND		NEG	-11	NEG	**NEG**
S29	ND		NEG	11	POS	**POS**
S30	ND		NEG	1	POS	**POS**
S31	*BRCA1:*Q1779Nfs*14	79%	POS	12	POS	**POS**
S32	ND		NEG	-5	NEG	**NEG**
S33	ND		NEG	14	POS	**POS**
S34	*BRCA1:*C1697R	84.2%	POS	13	POS	**POS**
S35	ND		NEG	-17	NEG	**NEG**
S36	ND		NEG	0	POS	**POS**
S37	ND		NEG	6	POS	**POS**
S38	*BRCA1:*Y1666*	65.4%	POS	3	POS	**POS**
S39	ND		NEG	-17	NEG	**NEG**

S40	ND		NEG	4	POS	**POS**
S41	ND		NEG	-18	NEG	**NEG**
S42	ND		NEG	-11	NEG	**NEG**
S43	ND		NEG	-13	NEG	**NEG**
S44	ND		NEG	-13	NEG	**NEG**
S45	ND		NEG	-5	NEG	**NEG**
S46	ND		NEG	-19	NEG	**NEG**
S47	ND		NEG	-10	NEG	**NEG**
S48	ND		NEG	-17	NEG	**NEG**
S49	*BRCA1:*C274*	82.7%	POS	25	POS	**POS**
S50	ND		NEG	-16	NEG	**NEG**
S51	ND		NEG	0	POS	**POS**
S52	ND		NEG	4	POS	**POS**
S53	ND		NEG	-12	NEG	**NEG**
S54	ND		NEG	-3	NEG	**NEG**
S55	ND		NEG	-13	NEG	**NEG**
S56	ND		NEG	18	POS	**POS**
S57	ND		NEG	5	POS	**POS**
S58	ND		NEG	8	POS	**POS**
S59	ND		NEG	-12	NEG	**POS**
S60	*BRCA2*:G2901Vfs*8	50.0%	POS	-19	NEG	**POS**
S61	ND		NEG	5	POS	**POS**
S62	ND		NEG	-16	NEG	**POS**
S63	ND		NEG	0	POS	**POS**
S64	ND		NEG	-20	NEG	**POS**
S65	*BRCA1:*G1348Nfs*7	62.7%	POS	5	POS	**POS**
S66	ND		NEG	4	POS	**POS**
S67	ND		NEG	10	POS	**POS**
S68	ND		NEG	4	POS	**POS**
S69	ND		NEG	-16	NEG	**NEG**
S70	ND		NEG	-13	NEG	**NEG**
S71	ND		NEG	-19	NEG	**NEG**
S72	*BRCA1:*Q541*	80.9%	POS	3	POS	**POS**
S73	*BRCA1:*Y1666*	63.6%	POS	7	POS	**POS**
S74	ND		NEG	7	POS	**POS**

Abbreviations: ND: not detected, NEG: negative, POS: positive, VAF: variant allele frequency

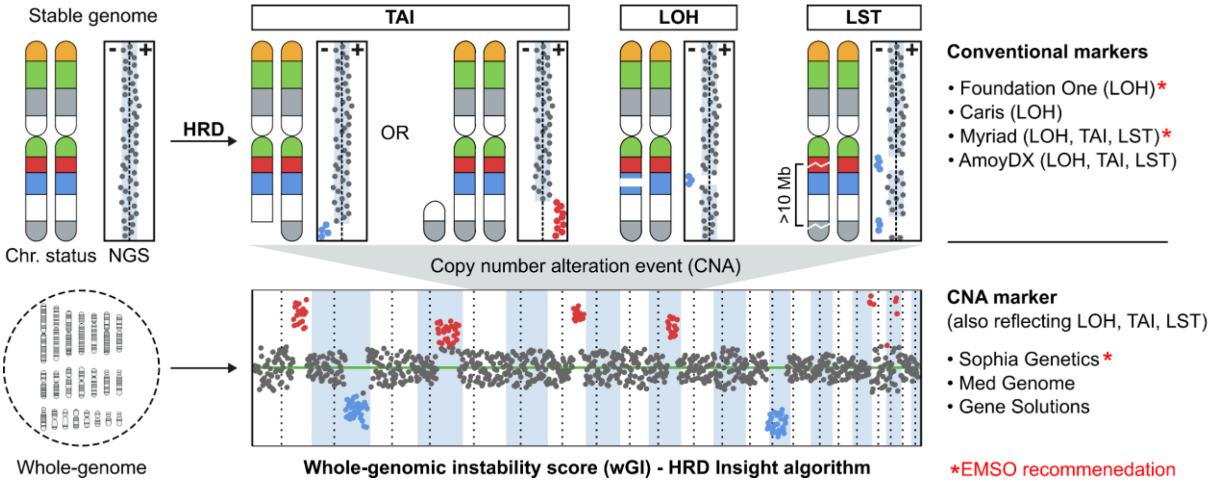

Figure S1. Biomarkers for homologous recombination repair deficiency (HRD). HRD in the tumor genome could result in chromosomal and subchromosomal abnormalities such as loss of heterozygosity (LOH), large-scale state transitions (LST) and telomeric allelic imbalance (TAI). Conventional markers of HRD including LOH, TAI, LST are examined in commercial HRD test kits from Foundation One, Myriad and AmoyDx. Copy number alterations (CNA) is a new biomarker that captures large genomic rearrangement events and also reflects LOH, TAI and LST occurrence. It has been examined in commercial HRD kits from Sophia Genetics, Med Genome and Gene Solutions.

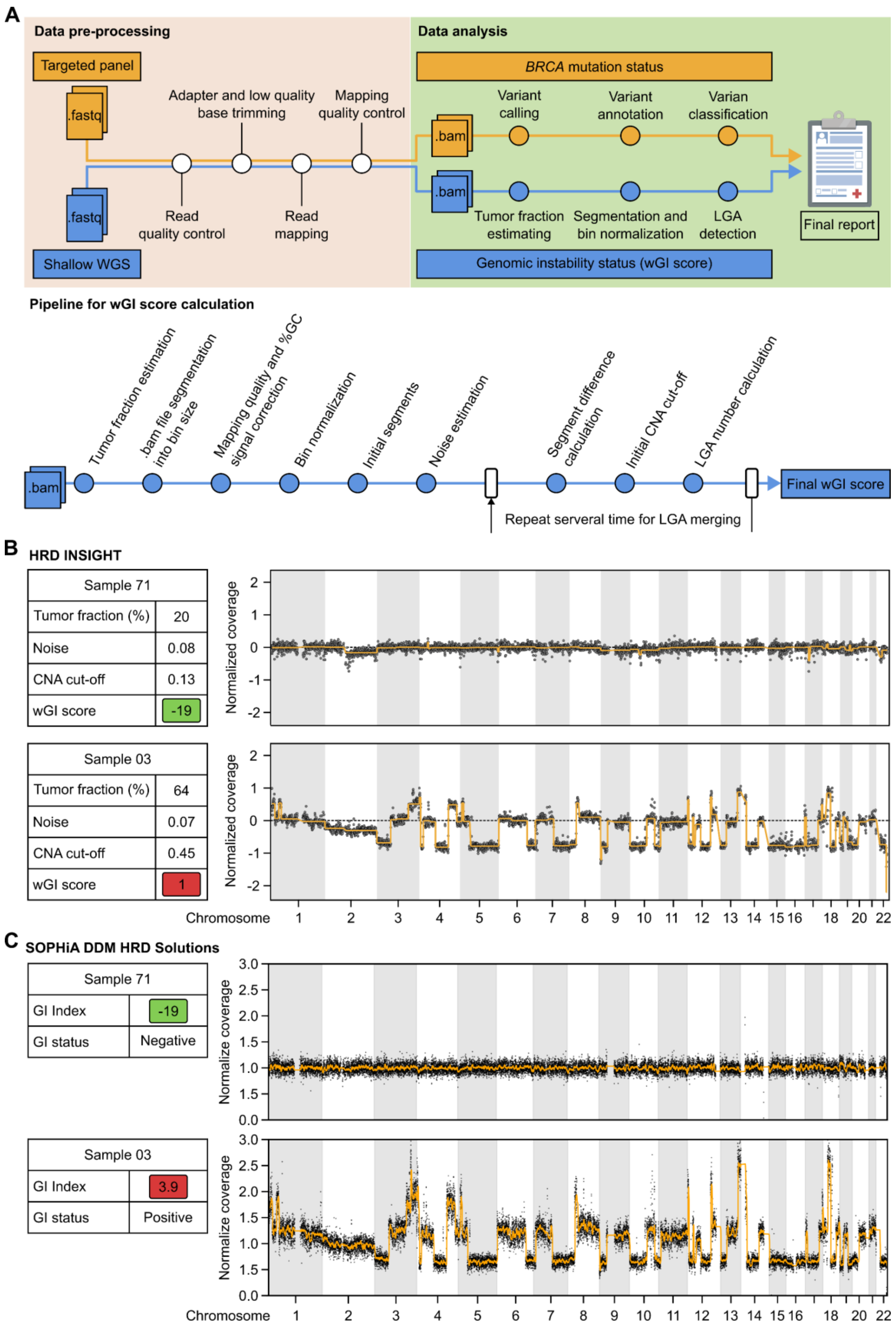

Figure S2. Data analysis workflow of the HRD INSIGHT assay. (A) The full pipeline illustrated data pre-processing steps including adapter removal, low-quality base trimming, reference genome mapping and QC; followed by analysis for *BRCA1/2* mutations and wGI score as described in the Methods. Representative outputs from the **(B)** HRD INSIGHT pipeline and **(C)** SOPHiA DDM HRD Solutions for samples with positive- and negative- wGI scores.

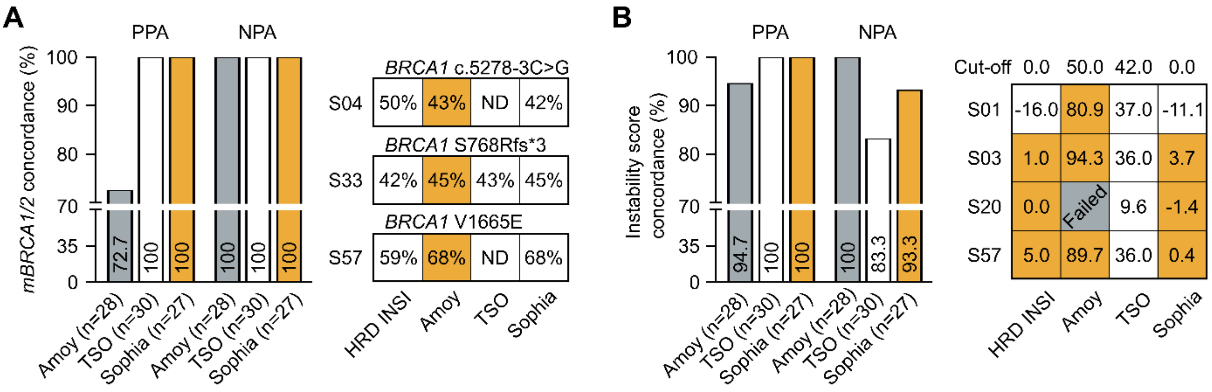

Figure S3. Analytical validation of the HRD INSIGHT assay. Negative- and positive- performance agreement (NPA and PPA) of the HRD INSIGHT assay with the HRD Focus Panel (AmoyDx), TruSight Oncology TSO 500 HRD (Illumina) and SOPHiA DDM HRD Solutions (Sophia Genetics) to determine **(A)** *BRCA1/2* mutation status and **(B)** genomic instability status of clinical FFPE ovarian samples. For conflicting samples, the results of individual assays were shown.

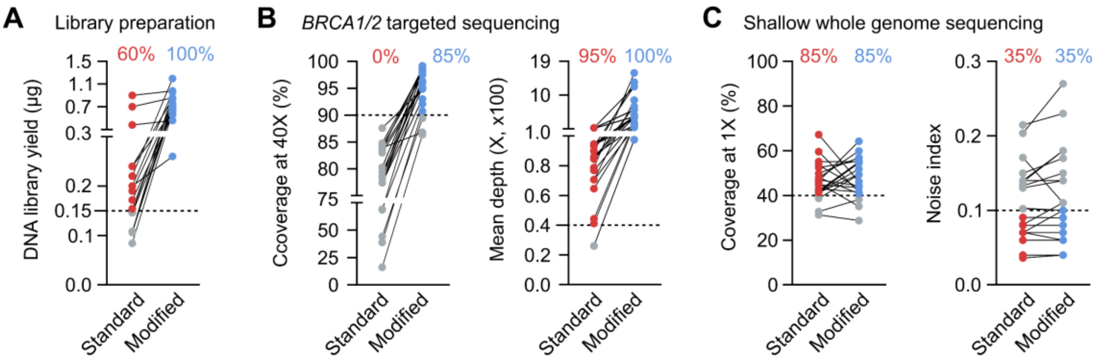

Figure S4. Modification of wet lab protocol improved library yield and successful rate for *BRCA1/2* targeted sequencing. A different set of in-house FFPE DNA samples (n = 20) was used to demonstrate the effectiveness of this modification. Using same DNA input, the DNA library yield **(A)** and QC measures for *BRCA1/2* targeted sequencing **(B)** were significantly improved for the modified protocol compared to the standard protocol. **(C)** QC for sWGS data were not different between the two protocols.
